# Microbiota-Modulating Strategies in Neonates Undergoing Surgery for Congenital Gastrointestinal Conditions: A Narrative Review

**DOI:** 10.3390/nu17132234

**Published:** 2025-07-05

**Authors:** Nunzia Decembrino, Maria Grazia Scuderi, Pasqua Maria Betta, Roberta Leonardi, Agnese Bartolone, Riccardo Marsiglia, Chiara Marangelo, Stefania Pane, Domenico Umberto De Rose, Guglielmo Salvatori, Giuseppe Grosso, Federica Martina Di Domenico, Andrea Dotta, Lorenza Putignani, Irma Capolupo, Vincenzo Di Benedetto

**Affiliations:** 1 Neonatal Intensive Care Unit, AOU Policlinico G. Rodolico San Marco, University of Catania, 95123 Catania, Italy; mlbetta@yahoo.it; 2 Pediatric Surgery Unit, Department of Medical and Surgical Sciences and Advanced Technologies, G. F. Ingrassia, AOU Policlinico G. Rodolico San Marco, University of Catania, 95123 Catania, Italy; mgscuderi@gmail.com (M.G.S.); vdbchirurgiapediatrica@gmail.com (V.D.B.); 3 Postgraduate Training Program in Pediatrics, Department of Clinical and Experimental Medicine, University of Catania, 95123 Catania, Italy; leonardi.roberta@outlook.it; 4 Postgraduate Training Program in Pediatric Surgery, Department of Medical and Surgical Sciences and Advanced Technologies, G. F. Ingrassia, AOU Policlinico G. Rodolico San Marco, University of Catania, 95123 Catania, Italy; agneseebartolone@gmail.com; 5 Unit of Microbiome, Bambino Gesù Children’s Hospital, IRCCS, 00165 Rome, Italy; riccardo.marsiglia@opbg.net (R.M.); chiara.marangelo@opbg.net (C.M.); 6 Unit of Microbiomics, Bambino Gesù Children’s Hospital, IRCCS, 00165 Rome, Italy; stefania.pane@opbg.net; 7 Neonatal Intensive Care Unit, Bambino Gesù Children’s Hospital, IRCCS, 00165 Rome, Italy; domenico.derose@opbg.net (D.U.D.R.); guglielmo.salvatori@opbg.net (G.S.); andrea.dotta@opbg.net (A.D.); irma.capolupo@opbg.net (I.C.); 8 Department of Biomedical and Biotechnological Sciences, University of Catania, 95123 Catania, Italy; giuseppegrosso82@gmail.com (G.G.); federica.didomenico@phd.unict.it (F.M.D.D.); 9 Department of Life Science, Health, and Health Professions, Link Campus University, 00165 Rome, Italy

**Keywords:** gut microbiota, newborns, gastrointestinal surgery, probiotics, prebiotics, postbiotics, lactoferrin, formula, breastfeeding, human milk

## Abstract

**Background/Objectives:** The gut microbiota (GM) is pivotal for immune regulation, metabolism, and neurodevelopment. Infants undergoing surgery for congenital gastrointestinal anomalies are especially prone to microbial imbalances, with a paucity of beneficial bacteria (e.g., Bifidobacteria and Bacteroides) and diminished short-chain fatty acid production. Dysbiosis has been associated with severe complications, including necrotizing enterocolitis, sepsis, and feeding intolerance. This narrative review aims to critically examine strategies for microbiota modulation in this high-risk cohort. **Methods:** An extensive literature analysis was performed to compare the evolution of GM in healthy neonates versus those requiring gastrointestinal surgery, synthetizing strategies to maintain eubiosis, such as early nutritional interventions—particularly the use of human milk—along with antibiotic management and supplementary treatments including probiotics, prebiotics, postbiotics, and lactoferrin. Emerging techniques in metagenomic and metabolomic analysis were also evaluated for their potential to elucidate microbial dynamics in these patients. **Results:** Neonates undergoing gastrointestinal surgery exhibit significant alterations in microbial communities, characterized by reduced levels of eubiotic bacteria and an overrepresentation of opportunistic pathogens. Early initiation of enteral feeding with human milk and careful antibiotic stewardship are linked to improved microbial balance. Adjunctive therapies, such as the administration of probiotics and lactoferrin, show potential in enhancing gut barrier function and immune modulation, although confirmation through larger-scale studies remains necessary. **Conclusions:** Modulating the GM emerges as a promising strategy to ameliorate outcome in neonates with congenital gastrointestinal surgical conditions. Future research should focus on the development of standardized therapeutic protocols and the execution of rigorous multicenter trials to validate the efficacy and safety of these interventions.

## 1. Introduction

The microbiota, the collection of symbiotic microorganisms (predominantly bacteria, but also Archaea, fungi, viruses, and protozoa) that live and interact with our body to regulate essential functions, represents “an organ within an organ”. The biological functions of the microbiota are mostly mediated by the production of short-chain fatty acids (SCFAs) [1]. Most microbiota microorganisms reside in the gut [2], the largest body surface exposed to the environment and pathogens, where 70% of immune cells reside. The composition of the intestinal microbiota thus acts as a dynamic barrier against pathogens, performs fundamental metabolic functions, and exerts a modulating action on the genome. Furthermore, SCFAs are involved in brain–gut axis cross-talk [3].

The development of a stable and healthy microbiota starts from the colonization that occurs in the newborn [4], according to gestational age, mode of delivery, and feeding type [5], up to adulthood [6]: alterations in the initial phase of colonization and/or in the maturation of the microbial flora ecosystem can lead to long-term consequences [7,8].

In newborns admitted to Neonatal Intensive Care Units (NICUs), especially those who undergo major surgery, gut microbiota composition is altered by several factors, such as antibiotic exposure, prolonged fasting, proton pump inhibitor use, total parenteral nutrition (TPN), invasive procedures, use of indwelling catheters and tubes, exposure to the NICU environment, and contamination by the hands of healthcare professionals. Dysbiosis of the gut microbiota in these patients has been associated with poor feeding tolerance, sepsis, cholestatic jaundice, and poor neurodevelopmental outcomes [9,10,11].

Strategies to maintain gut eubiosis in this cohort of children are indeed of utmost importance.

The aim of this narrative review was to analyze which strategies have shown efficacy in modulating gut microbiota composition in newborns undergoing major surgery for congenital gastrointestinal morbidities.

## 2. Materials and Methods

### 2.1. Search Strategy

This review followed the guidelines of the Scale for the Assessment of Narrative Review Articles (SANRA) [12]. A literature search was performed across PubMed, Scopus, Web of Science, and Google Scholar to identify relevant studies published between 2000 and 2024. The search used Boolean operators to combine terms such as “newborn”, “gastrointestinal surgery”, “enteral nutrition”, “parenteral nutrition”, “gut microbiota”, “probiotics”, “prebiotics”, “postbiotics”, “lactoferrin”, “human milk”, “antibiotics”, “proton pump inhibitors”, and “formula milk”. Additional sources were identified through a manual search of reference lists in relevant articles.

### 2.2. Eligibility Criteria

Studies were selected based on predefined inclusion and exclusion criteria.

Randomized controlled trials (RCTs), observational studies, meta-analyses, and systematic reviews were included in the analyses. Clinical guidelines and expert recommendations were also considered. Only articles published in English were evaluated. Exclusion criteria comprised (1) articles published in languages different from English, (2) pre-clinical and animal studies, (3) case reports, editorials, and conference abstracts with insufficient data. The level of evidence was assigned according to the classification of the Oxford Centre for Evidence-Based Medicine (2011), “The Oxford Levels of Evidence 2” (OCEBM Levels of Evidence Working Group*. Oxford Centre for Evidence-Based Medicine https://www.cebm.ox.ac.uk/resources/levels-of-evidence/ocebm-levels-of-evidence; accessed on 2 May 2025).

### 2.3. Study Selection Process

Three reviewers (N.D., F.DD., and R.L.) performed the data search and analysis. The selection of studies was guided by their relevance to the review’s objectives rather than by the systematic inclusion criteria; the SANRA checklist was used to assess strengths and weaknesses [12]. Titles and abstracts were initially screened, followed by full-text examination. Studies on adults and those focusing on newborns not affected by gastrointestinal surgical conditions were evaluated for background and commentary.

### 2.4. Data Synthesis

Studies were narratively synthesized.

An overview of gut microbiota dynamics in healthy term newborns and in newborns affected by gastrointestinal surgical conditions was included to help specify the problem and the relevance of the study aim. Key study arguments (e.g., use of prebiotics, probiotics, postbiotics, lactoferrin) were extracted to organize several thematic sections. This approach facilitated the synthesis of heterogeneous evidence.

## 3. Results

### 3.1. Dynamics of the Microbiota in a Healthy Term Newborn

Recent studies have highlighted the presence of bacteria in amniotic fluid, placenta, and meconium, revealing that the fetus in utero does not grow in a sterile environment, but a mother-to-fetus microbial transfer occurs during pregnancy [13,14]. Nevertheless, the most important phase of gut colonization in newborns occurs after birth, and the delivery mode and the initial nutrition play a pivotal role [15].

In a term infant born via spontaneous vaginal delivery, the first colonizing species in the intestine are similar in composition to the maternal vaginal microbiota [16], dominated by *Lactobacillus*, *Prevotella*, and facultative anaerobes such as *Escherichia* spp. and other members of the Enterobacteriaceae family [17]. Conversely, term infants born via cesarean section have an initial intestinal colonization dominated by strains present on the maternal skin and the surrounding environment: *Staphylococcus*, *Corynebacterium*, and *Propionibacterium* [18]. Colonization by *Bifidobacteroides* and *Bifidobacterium* is delayed by about a month after birth compared to vaginally born infants due to the administration of antibiotic prophylaxis to the mother and the delayed onset of breastfeeding (especially in planned cesarean sections) [19,20,21].

In the first days of life, diet plays a key role in the development of colonizing bacterial species [22]. Exclusively breastfed infants have a more uniform population of microbes, with a prevalence of *Bifidobacterium*, *Bacteroides*, and *Clostridium* spp. [23]. Bacterial transfer from the mother to the breastfed child depends on the transfer of bacteria on the mother’s skin during suckling (e.g., *S. mitis*, *S. salivarius*, and coagulase-negative Staphylococci), but the entero-mammary pathway hypothesis states that major maternal intestinal bacteria (e.g., *Bacteroides* and Clostridia) may reach the mammary glands via maternal dendritic cells and macrophages [24,25,26]. This could explain the same bacterial species found in the human milk (HM) and feces of mother–child dyads [27], influenced by breastfeeding exclusivity and duration and less by feeding mode (suckling vs. pumping) [28].

A significant component of HM influencing neonatal gut colonization is represented by HM oligosaccharides (HMOs) such as galacto-oligosaccharides (GOSs) and fructo-oligosaccharides (FOSs) [29,30]. HMOs are not digested in the small intestine of the newborn and reach the colon intact, where they serve as nourishment for Bifidobacteria and *Bacteroides*, which produce an enzyme, lacto-N-biosidase, that digests HMOs [29]. These bacteria limit the development of other colonies through acidification of the environment [31], since the metabolism of HMOs results in the production of lactate and SCFAs [30,32]. Bifidobacteria, in particular the *Bifidobacterium infantis*, have also been associated with Immunoglobulin A in the gut, thus contributing to neonatal immunity [33].

The gut microbiota of formula-fed infants indeed shows a decreased quantity of bacteria, with an increased diversity and fewer Bifidobacteria compared to breast-milk-fed infants. The predominance of Staphylococci, *Bacteroides*, Clostridia, Enterococci, Enterobacteria, and the genus *Atopobium* [34] in the microbiota of formula-fed infants is due to the absence of HMOs and a higher amount of protein content in formula milk (FM), which determines a proteolytic metabolism. This produces compounds that can be converted into pro-inflammatory metabolites in the liver and are associated with increased gut permeability, such as p-cresol-sulfate and phenylacetate-glutamine. This pattern is not completely reversed by the addition of GOSs and FOSs to infant formulas, because they are less selective than HMOs [35], nor by FM supplemented with probiotics [36].

### 3.2. Dynamics of the Microbiota in the Surgical Newborn

The main congenital gastrointestinal conditions requiring surgery in the neonatal period (congenital gastrointestinal surgical conditions, CGISCs) include wall defects (gastroschisis and omphalocele), duodenal atresia, small and large intestinal atresia, esophageal atresia, malrotation and volvulus, meconium ileus, left colon hypoplasia, Hirschsprung’s Disease (HD), anorectal malformations, and congenital diaphragmatic hernia (CDH). Necrotizing enterocolitis (NEC), although not congenital, is another frequent condition requiring surgery in the newborn. All of these conditions are associated with feeding intolerance and increased infection risk [37]. Furthermore, these children present with a series of risk factors that expose them to early and prolonged alterations of the intestinal microbiota, such as the need for hospitalization in NICU, delayed initiation of enteral nutrition, limited use of mother’s milk, and the use of invasive procedures and devices. These children are exposed to prolonged courses of antibiotic therapy, parenteral nutrition, and gastric acid suppressors [38].

The situation is further complicated when preterm birth occurs, as there is often a maternal history of chorioamnionitis and maternal antibiotic use, in addition to the intestinal and immune immaturity of the child [39]. The presence of bacteria in the amniotic fluid may lead to amniositis, funisitis, and chorioamniositis, which are commonly linked to preterm delivery [40].

In a recent prospective study, Rao et al. compared the intestinal microbiota of a group of neonates with CGISCs at a gestational age (GA) of >36 weeks with the microbiota of a group of healthy neonates of the same age [41]. CGISC neonates exhibited significant intestinal dysbiosis as early as in the second week of life, with a deficit of eubiotic bacteria such as *Bacteroides* and *Bifidobacterium* and an overabundance of *Escherichia–Shigella* and *Pseudomonas*. Additionally, while healthy neonates showed a significant increase in the production of SCFAs in the first few weeks of life, these were markedly reduced in the feces of CGISC neonates (Table 1).

The primary determinant of these differences seemed to be diet. Neonates with CGISCs often undergo delayed enteral feeding and prolonged TPN periods. TPN is associated with a persistent reduction in the ecological parameters of the gut microbiota, defined by the richness and diversity of the colonizing species within the first 4 weeks of life; furthermore, neonates receiving TPN have a lower abundance of Bacteroidetes compared to healthy controls and a higher abundance of Verrucomicrobia and Proteobacteria [42]. This can have important long-term consequences for immunity and resistance to pathogen colonization.

The capsular polysaccharide A of the genus *Bacteroides* activates IL-10 and reduces the activation of the innate immune response by increasing the production of secretory IgA and regulating the action of T-helper lymphocytes. In vitro, secretion products of *Bifidobacteria* downregulate the production of the pro-inflammatory cytokines IL-8 and IFNγ and enhance the anti-inflammatory pathway by increasing IL-10 production. Acetate production by Bifidobacteria protects against pathogen colonization. Neonates on TPN have also been found to have an increased growth of *Akkermansia muciniphila*, a strain that degrades mucin and can survive in nutrient-scarce environments, such as during prolonged fasting and malnutrition. Although this strain has been associated with beneficial effects in contexts such as obesity and type I diabetes [43], and its absence or reduction has been identified as an indicator of local and systemic inflammation [44], other studies correlate the overgrowth of *Akkermansia* with thinning and loss of barrier functions of the intestinal epithelium, which promotes the intestinal translocation of pathogens, inflammation, and colorectal cancer [45].

A reduction in the richness of the intestinal microbiota, a scarcity of *Bacteroides*, and an abundance of Proteobacteria have also been highlighted in children developing NEC [8,11] and TPN-related cholestasis [46]. Prolonged broad-spectrum antibiotic therapies and the use of proton pump inhibitors also contribute to creating a similar dysbiotic profile. McDonnell’s meta-analysis reveals that antibiotic use is associated with a reduction in the richness and diversity of the intestinal microbiota, with macrolides associated with such alterations for twice as long as penicillin [9]. In terms of taxonomic composition, the use of ampicillin, cephalosporins, gentamicin, and macrolides is associated with a reduction in *Bifidobacterium*, *Bacteroides*, and *Lactobacillus* and the growth of *Clostridium* spp. and *Escherichia coli*, the latter being an important cause of urinary tract infections and sepsis and a mediator of antibiotic resistance.

Finally, an antibiotic therapy duration > 5 days and a broad spectrum of action are associated with invasive fungal infections. In a perfect vicious circle, intestinal dysbiosis thus favors the onset of infection due to the translocation of multidrug-resistant pathogens [47]. This problem is of particular importance for neonates with Short Bowel Syndrome (SBS), where bloodstream infections (BSIs) and TPN-associated liver disease (PNALD) represent the main cause of mortality. In SBS, both complications are determined by bacterial overgrowth in the residual intestine, which increases in size and has reduced peristalsis, leading to these children periodically receiving cycles of antibiotic therapy that further aggravate intestinal dysbiosis. The intestinal microbiota of children with SBS is characterized by reduced microbial richness and diversity, with a prevalence of Enterobacteriaceae and a depletion of obligatory anaerobes producing SCFAs, resulting in an increased risk of infections and poor growth [10,48].

Indeed, neonates with CGISCs are also treated with drugs that reduce stomach acid secretion, such as proton pump inhibitors (PPIs) and H2-receptor antagonists (H2RAs), used during prolonged fasting and in the management of gastroesophageal reflux disease, which complicates many of the conditions under analysis. These drugs favor the onset of NEC and late-onset sepsis (LOS) through a mechanism related, on the one hand, to the dysbiosis of the intestinal microbiota, as gastric acidity prevents the growth of pathogens, and, on the other hand, to an immunological effect of blocking the activity of basophils and mast cells [49,50].

### 3.3. Strategies to Modulate the Intestinal Microbiota in Newborns Undergoing Gastrointestinal Surgery

#### 3.3.1. Early Enteral Feeding

The early initiation of enteral nutrition in surgical children was recently evaluated through a review trial that included 488 patients aged between 10 days and 6.5 years [51]. An intervention group of 217 children received enteral nutrition within 8 h and 48 h after surgery (early enteral nutrition—EEN), while a control group of 271 children received enteral nutrition 48 h after surgery (late enteral nutrition—LEN). The results of the meta-analysis showed that early-fed children had shorter hospital stays, quicker resumption of bowel movements, and reduced surgical wound infections, without differences in suture dehiscence, vomiting, or abdominal distension. The same results were reported in a recent meta-analysis by Behera et al. who evaluated 1286 children from 10 studies who underwent EEN or LEN after bowel anastomosis [52] (Table 1). The availability of internal protocols defining criteria for initiating and stopping enteral feeding, modes of administration, and advancement standards has been demonstrated to improve the time to reach full enteral feeding and nutritional outcomes after gastrointestinal surgery [53,54].

HM should be the first choice, if available. In a trial conducted on patients with gastroschisis, feeding with HM reduced the time to reach full enteral feeding and hospital discharge [55] (Table 1). In the absence of the mother’s own milk, donor HM (DHM) should be the second choice, especially if the neonate is prematurely born, as this provides essential nutritional and bioactive components, although we have no randomized controlled trials reporting the superiority of DHM versus formulas in the refeeding after major surgery. DHM helps prevent key prematurity complications, particularly NEC up to 36 weeks postmenstrual age [56,57], and shows better tolerance.

**Table 1:** Summary of relevant studies regarding gut microbiota ecology in neonates with congenital gastrointestinal surgical conditions and potential related modulation algorithms.

N.	Author, Year	Type of Study	Level of Evidence	Age	Number of Patients	Main Topic	Key Findings	Reference
1	Rao SC et al., 2020	Prospective Cohort Study	Level II	Newborns (≥36 weeks)	73	Gut microbiota composition in neonates with CGISCs vs. healthy infants	By the second week of life, neonates with congenital gastrointestinal surgical conditions (CGISCs) developed gut dysbiosis, characterized by a lower abundance of *Bifidobacterium* and *Bacteroides* and higher levels of *Pseudomonas* and *Escherichia–Shigella* compared to healthy infants. Stool SCFA levels were significantly lower in CGISCs. Findings suggest the potential for probiotic interventions.	[41]
2	Issac A et al., 2023	Systematic Review and Meta-analysis	Level I	10 days–6.5 years	488	Early enteral nutrition (EEN) vs. late enteral nutrition (LEN)	Early enteral nutrition (EEN) after gastrointestinal surgery showed shorten hospital stays, faster recovery by promoting earlier fecal movement, and reduced postoperative wound infections. No significant impact on anastomosis leakage, vomiting, or abdominal distension.	[51]
3	Behera BK et al., 2022	Systematic Review and Meta-analysis	Level I	0–18 years	286	EEN vs. LEN in children following bowel anastomosis surgery	Early enteral nutrition after bowel anastomosis surgery showed a statistically significant lower incidence of surgical site infections, septic complications, and pooled overall complications compared to LEN group. The time to the passage of first feces and the length of hospitalization were significantly lower in the EEN group. No significant difference in anastomotic leaks, abdominal distension, wound dehiscence, or vomiting between EEN and LEN.	[52]
4	Kohler JA et al., 2013	Retrospective Study	Level III	Newborns	90	Exclusive HM vs. formula feeding after gastroschisis repair	Exclusive HM feeding led to significantly shorter time to full enteral feeds and hospital discharge compared to formula-fed infants. Findings suggest HM should be prioritized in post-gastroschisis feeding strategies.	[55]
5	Brindle ME et al., 2013	Consensus Based Guidelines	Level I	Newborns (≥37 weeks)	Not Applicable	ERAS recommendations for perioperative care in neonatal intestinal surgery	Seventeen evidence-based recommendations for Enhanced Recovery After Surgery (ERAS) in neonatal intestinal surgery. Key points include early enteral feeding within 24–48 h, prioritization of h milk, perioperative fluid management, prevention of hypothermia, structured perioperative communication, and parental involvement in care.	[58]
6	Rao SC et al., 2018	Systematic Review	Level I	Newborns	32	Probiotic use, gut microbiota composition, clinical outcomes in infants with gastrointestinal surgical conditions	Limited evidence on probiotics in neonates with gastrointestinal surgical conditions. Two RCTs (N = 32) evaluated probiotic supplementation in neonates with GI surgical conditions. One RCT (N = 24) found no significant differences in overall microbial composition, though probiotics increased Bifidobacteriaceae and reduced Clostridiaceae, Enterobacteriaceae, Enterococcaceae, Staphylococcaceae, and Streptococcaceae. Another RCT (N = 8) showed increased Streptococcaceae in the probiotic group but unexpectedly higher Bifidobacteriaceae in the control group. No significant differences in TPN duration, antibiotic therapy, or length of hospital stay were observed. Surgical stress appears to significantly affect gut microbiota.	[59]
7	Powell WT et al., 2016	Randomized Controlled Trial (RCT)	Level I	Newborns (>34 weeks)	24	Probiotic use, fecal microbiota composition, hospital stay	*Bifidobacterium infantis* probiotic altered gut microbiota composition but had no significant impact on length of hospital stay.	[60]
8	Murakami et al., 2016	Randomized Controlled Trial (RCT)	Level I	Newborns (≥37 weeks)	13	Probiotic use, fecal microbiota composition	The authors randomized 8 CGISC patients; 4 received *Bifidobacterium animalis* subsp. lactis LKM512 (LKM) and 4 received placebo; 3 healthy newborns served as controls. Stool analysis revealed similar results for both groups; particularly, there were more Streptcoccaceae in stools from those who received probiotics and, unexpectedly, there were more Bifidobacteriaceae in samples from those who did not receive probiotics. The authors concluded that stress after surgery has an important impact on GM.	[61]
9	Rao SC et al., 2022	Randomized Controlled Trial (RCT)	Level I	Newborns (>35 weeks)	61	Gut microbiota, SCFA levels, clinical outcomes	Probiotic supplementation in neonates with CGISC significantly reduced the relative abundance of pathogenic bacterial families (*p* = 0.044) and increased Bifidobacteriaceae levels (*p* < 0.001) at two weeks. SCFA levels were higher in the probiotic group (*p* = 0.008), with no significant differences in hospital stay, infections, or antibiotic use. Postnatal head circumference restriction was less severe in the probiotic group (*p* = 0.013). No probiotic-related infections were reported, supporting safety and potential benefits for gut microbiota modulation.	[62]
10	Trivedi A et al., 2024	Systematic Review	Level I	Newborns (>35 weeks)	61	Use of probiotics after gastrointestinal surgery for postoperative management	Probiotics showed little to no effect on the incidence of proven sepsis (OR 0.64, 95% CI 0.16–2.55) or time to full enteral feeds (MD 0.63 days, 95% CI −4.02 to 5.28). No deaths were reported before hospital discharge. After two weeks of supplementation, infants receiving probiotics had a significantly higher abundance of beneficial intestinal microflora (Bifidobacteriaceae) compared to the placebo group.	[63]
11	Trivedi A et al., 2023	Systematic review	Level I	Newborns (>37 weeks)	Not applicable	Lactoferrin for postoperative management after gastrointestinal surgery	No RCTs were identified evaluating the efficacy of lactoferrin in postoperative term neonates after gastrointestinal surgery. There is no current evidence to support or refute its use in reducing sepsis, mortality, or improving enteral feeding.	[64]

Abbreviations: CGISCs: congenital gastrointestinal surgical conditions; SCFA: short-chain fatty acid; EEN: early enteral nutrition; HM: human milk; LEN: late enteral nutrition; ERAS: Enhanced Recovery After Surgery; RCTs: randomized controlled trials; TPN: total parenteral nutrition. The level of evidence has been assigned according to the classification of the Oxford Centre for Evidence-Based Medicine (2011).

#### 3.3.2. Reducing the Duration of Antibiotic Therapy

Antibiotics are among the main drugs used in neonatology. Despite this, there is wide variability among NICUs regarding protocols in use, drug choice, dosage, and treatment duration. Given the impact of antibiotics on altering the intestinal microbiota, reducing their use is of fundamental importance [9,47,48,65]. Riddle et al., evaluating newborns with gastroschisis, demonstrated that before surgery, most of them had received prophylactic antibiotic therapy at birth, despite the low incidence of early-onset sepsis (EOS) [66]. Implementing antibiotic stewardship protocols is essential to avoid unnecessary antibiotic prophylaxis and reduce antibiotic resistance and prolonged alteration of the intestinal microbiota [67]. Such protocols should balance the risk of unnecessary antibiotic exposure with the risk of post-surgery infections.

The incidence rate of surgical site infection (SSI) among neonates and infants is reported to be between 0.7 and 16.6%, with high morbidity and prolonged length of hospital stay [68,69,70].

Recently, the Enhanced Recovery After Surgery (ERAS^®^) Society recommendations on perioperative care in neonatal gastrointestinal surgery suggested providing antibiotic prophylaxis within 60 min before surgical incision, in line with recommendations from adult and pediatric patients 1–18 years undergoing clean–contaminated or contaminated abdominal gastrointestinal operations [58,71]. A single-dose prophylaxis or a duration < 24 h regardless of the presence of intravascular catheters or indwelling drains is recommended [71]. Postoperative antibiotic treatment duration in newborns and infants lacks robust evidence and is often prolonged due to concerns over neonatal immunodeficiency and the high mortality rate of neonatal severe infections. In a few studies, reducing the duration of post-surgical therapy to 72 h did not increase the incidence of LOS or mortality [72,73,74] and is, therefore, a practice to be encouraged [58].

#### 3.3.3. Probiotics

Probiotics are live microorganisms useful in modulating the composition of the intestinal microbiota. The probiotics most frequently utilized in newborns and infants, commonly as an additive to infant FM, are predominantly selected based on their safety profile, ability to colonize the gastrointestinal tract, and specific health-promoting effects [75]. These include species from the genus Bifidobacterium (e.g., Bifidobacterium breve, Bifidobacterium infantis) [76,77] and the genus Lactobacillus (e.g., Lactobacillus rhamnosus, Limosilactobacillus reuteri or Lactobacillus reuteri, Lactobacillus acidophilus) [78,79,80]. Numerous studies conducted on neonates show that probiotics prevent bacterial overgrowth, reduce the risk of infections and NEC, promote barrier functions and intestinal motility [81], and have preventive potential against multidrug-resistant organisms (MDRO+) or highly epidemic bacteria [80]. Probiotic supplementation has demonstrated efficacy in restoring microbiota balance disrupted by antibiotic exposure or other perinatal factors in both term and preterm populations [82,83,84]. Moreover, when breastfeeding is not possible, exposure to some probiotics could modify the microbiota composition [49]. Strains from Bifdobacterium and Lactobacillus contribute to healthier gut microbiota, enhance gut barrier function, and modulate immune responses in preterm infants [85]. Multi-strain probiotic formulations, such as Lactobacillus acidophilus in combination with Bifidobacterium bifidum, produce superior outcomes compared to single-strain formulations, with significant reductions in the incidence of NEC [86,87,88]. The multi-strain combination of Bifidobacterium infantis, Bifidobacterium lactis, and Streptococcus thermophilus led to similar results in some studies, although other trials were underpowered to detect significant differences [87,89,90].

Data on adults undergoing gastrointestinal surgery demonstrate the efficacy of probiotics, alone or in combination with prebiotics, in reducing the incidence of SSIs, sepsis, and urinary tract infections (UTIs) [91,92,93].

Evidence on probiotic use in CGISC neonates is still limited, though promising [59,94].

Three randomized controlled trials (RCTs) tested the effectiveness of different strains of Bifidobacteria and Lactobacillus in surgical neonates, demonstrating a lower presence of enteropathogens in treated children. Powell et al. randomized 24 newborns with gastroschisis > 34 weeks GA to receive *Bifidobacterium infantis* or placebo twice daily. Analysis of the final specimens showed higher Bifidobacteriaceae and lower Clostridiaceae, as well as trends toward lower Enterobacteriaceae, Enterococcaceae, Staphylococcaceae, and Streptococcaceae, in the probiotic group. Clinical outcomes, including length of hospital stay, did not differ between the groups [60]. Murakami et al. randomized eight CGISC patients, four to receive *Bifidobacterium animalis* subsp. lactis LKM512 (LKM) and four to receive placebo; three healthy newborns served as controls. There were more Streptcoccaceae in stools from those who received probiotics and, unexpectedly, there were more Bifidobacteriaceae in the samples from those who did not receive probiotics. The authors concluded that stress after surgery has an important impact on GM and that probiotic use deserves more studies [61]. In a randomized pilot trial, Rao et al. randomized 61 neonates with CGISCs to receive a daily triple-strain bifidobacterial probiotic or placebo. Neonates who received probiotics demonstrated a lower number of potentially pathogenic families of bacteria in the stool and a higher relative abundance of *Bifidobacteriaceae* compared to the placebo group [62] (Table 1).

A recent Cochrane review analyzed one RCT including 61 newborns ≥ 35 weeks treated with probiotics for postoperative management after GI surgery. Probiotics increased the abundance of beneficial bacteria in the stools of treated newborns, but efficacy in terms of proven sepsis or time to full enteral feeds needs to be evaluated in adequately powered RCTs [63] (Table 1).

There are some limitations to the use of probiotics in clinical practice.

First of all, the risk of probiotic-induced bacteremia is low, but not negligible [95], although most published cases of probiotic sepsis were successfully treated because of the low pathogenicity of the organisms involved (especially Bifidobacterium species) and good sensitivity to standard antibiotics [96].

The second limit is that colonization by probiotics is not stable over time, and it is crucial that supplementation occurs early, within the first month of life [22].

Further research is needed, with well-designed multicenter RCTs on probiotic supplementation for neonates with CGISCs, which must have certain characteristics to obtain the answers we need [97] (Table 2).

#### 3.3.4. Prebiotics

Prebiotics are non-digestible substances contained in some foods (such as non-starch polysaccharides) that promote the growth of beneficial bacterial species in the colon [98]. In neonatology, the most used prebiotics are GOSs and FOSs, which are added to FM for their similarity to HMOs. GOSs and/or FOSs added to FM promote the growth of Bifidobacteria and reduce fecal pH [31]. Prebiotic enrichment in breastfeeding can enrich the Lactobacilli component of the gut microbiota of newborns [99]. Research has indicated that prebiotic supplementation may contribute to a reduction in the incidence of gastrointestinal infections, facilitate gut colonization by beneficial microbial populations, and enhance the maturation of the infant immune system [100,101]. The impact of prebiotics alone on reducing NEC is non-homologous among studies [102,103]. RCTs on prebiotic administration in preterm newborns were systematically reviewed by Srinivasjois et al. Prebiotics did not reduce the incidence of NEC, LOS, or time to full enteral feeding, but they were safe and increased the amount of beneficial bacteria in stool [104]. Better outcomes are associated with the use of synbiotics. A synbiotic refers to a formulation comprising live microorganisms and substrates that are selectively metabolized by host microorganisms to confer a health benefit [105]. Synbiotics are categorized as either complementary, where the probiotic and prebiotic independently contribute to health outcomes, or synergistic, where the prebiotic is specifically selected to enhance the growth and activity of the probiotic [106]. In a double-blind, randomized trial conducted by Mahboobipour et al., a synbiotic containing probiotics and prebiotics was effective in reducing the incidence of NEC and feeding intolerance in a group of very-low-birth-weight (VLBW) infants compared to those receiving placebo [107]. In a randomized trial conducted by Dilli et al., NEC incidence was lower in groups receiving probiotics and synbiotics compared to those receiving prebiotics or placebo [108]. There are no data evaluating the efficacy of prebiotics or synbiotics in newborns affected by CGISCs.

#### 3.3.5. Postbiotics

Postbiotics are inactivated microorganisms or their components/metabolites (extracts) capable of conferring a benefit to the host [109]. Those molecules specifically encompass cellular components such as cell wall structures, including peptidoglycans and lipoteichoic acids; metabolic byproducts, including lactic acid and SCFAs; and bioactive molecules, such as bacteriocins and biosurfactants [110]. Postbiotics have a trophic function in the intestinal mucosa by stimulating mucosal immune responses and exert an immunomodulating effect by interacting with immune receptors (e.g., Toll-like receptors, TLRs) to regulate inflammatory responses [111]. A potential immunomodulatory mechanism of postbiotics is illustrated by an in vitro study examining the innate response of macrophages to non-viable *Lactobacillus casei* cells. Exposure to a suspension containing heat-killed bacterial cells led to an upregulation in the expression of pro-inflammatory cytokines and an enhanced transcription of TLR-2, TLR-3, TLR-4, and TLR-9 [112]. As research exploring these molecular mechanisms is often conducted in vitro and in vivo, the precise pathways responsible for these observed benefits in humans remain incompletely understood [113]. Currently, given the various species-specific properties, the significant heterogeneity of the analyzed substrates, and the great variability of potentially interfering conditions, there are no clear indications for their use in neonatal clinical practice [109]. Limited evidence from a systematic review suggests that infants consuming FM enriched with postbiotics—produced mainly through fermentation by strains such as *Bifidobacterium breve* and *Streptococcus thermophilus*—exhibited a gut microbiota composition more closely resembling that of breastfed infants. Additionally, these FMs may enhance immune responses, as indicated by elevated levels of stool secretory IgA, alongside with a reduction in gastrointestinal symptoms and infection rates [114]. Furthermore, findings from a systematic review and meta-analysis indicate that postbiotic-enriched FM does not increase the incidence of serious adverse events (SAEs) or gastrointestinal complications [115]. In a recent prospective observational cohort study conducted on late preterm infants, De Bernardo et al. revealed distinct metabolomic differences between breastfed and formula-fed infants, emphasizing the potential of postbiotic-enriched formula from *Lactobacillus paracasei* to replicate the benefits of HM. Postbiotic-enriched formula more closely approximated the metabolomic patterns observed in breastfed infants [116]. Regardless, the investigation of postbiotics remains in its nascent stages, as most studies are preliminary or focused on animal models, while human studies often lack methodological standardization and strain-specific outcomes, resulting in limited clinical evidence to robustly validate the efficacy of postbiotics in the prevention or management of CGISCs [117].

#### 3.3.6. Lactoferrin

Lactoferrin (Lf), a serum glycoprotein, is a member of the transferrin family secreted by epithelial cells and polymorphonuclear cells, highly concentrated in colostrum and HM.

Lf exerts antimicrobial, anti-inflammatory, and immunomodulating activities [118,119,120], which are dependent on and independent of its iron-binding capacity. Indeed, iron is important for bacterial and fungal growth and biofilm formation and is involved in oxidative stress-induced reactions. Lf binds two ferric ions per molecule, thus creating an iron-deficient environment. Furthermore, Lf exerts a direct bactericidal effect on both Gram-negative and Gram-positive bacteria through the binding to membrane lipopolysaccharides (LPS) and lipoteichoic acid [121] and reduces the cellular internalization of bacteria and viruses via competitive binding to host cell receptors and/or pathogen particles [122].

The administration of bovine Lf (bLf), which shares over 70% homology with human LF, reduces the incidence of LOS in preterm infants [123,124,125,126] alone or in combination with probiotics, as confirmed in a recent meta-analysis by Razak et al., which included 3679 infants [127]. Data on Lf’s efficacy in reducing NEC and mortality are, however, conflicting [128,129]. In a follow-up study on 743 VLBW newborns, Manzoni et al. demonstrated that bLf could reduce the incidence of death and/or NEC compared to placebo. These data were not confirmed in two large, randomized trials, the ELFIN trial (2203 infants enrolled in the UK) [130] and the LIFT trial (1542 infants enrolled in Australia) [131]. A meta-analysis including 5609 preterm infants confirmed that Lf supplementation could significantly reduce LOS in preterm infants, but not NEC or all-cause mortality. As argued by the authors, the use of different commercial products with different LF concentrations, saturation levels, and chelating properties may have influenced the results in the different trials, considering that the antibacterial efficacy of lactoferrin can be reduced by 40–70% depending on differences in bovine vs. human Lf and in storage and processing techniques [132]. The type of enteral feeding associated with Lf supplementation (HM or FM) could also have influenced trial results and deserves further research [133]. Lf can modulate the microbiota, as recently reported by D’Amico et al. in an RCT conducted on pediatric patients undergoing induction chemotherapy and randomized to receive Lf 200 g/die or placebo (Table 1). Microbiota in Lf-treated patients conserved alpha diversity and showed a larger amount of eubiotic bacteria and a reduced amount of Enterobacteria and *Akkermansia*, compared to the placebo group [134]. There are no specific studies on the LF use in neonates with CGISCs, but this population of patients would benefit from its antimicrobial and modulating properties [64].

### 3.4. The Impact of Microbiome Analytics: A New Approach in Clinical Microbiology to Decipher Neonatal Pathological Conditions from the Laboratory Point of View

During the last decade, a growing number of high-throughput technological platforms and functionality analyses have been exploited to study the gut microbiota, overcoming the usual limits imposed by classical microbiology and improving the analytical performance for viable but uncultivable microbial components.

These high-throughput molecular-based techniques have opened new avenues in the knowledge of the taxonomic and phylogenetic framework of the intestinal microbiota. Such novel techniques have added further knowledge on the intestinal ecosystem’s complexity and on the identification of previously uncategorized bacterial populations. However, the complexity of this system requires a “systems biology” approach, able to describe not only components and sets of components but also complex biological networks across multiple hierarchical levels [135].

**Figure 1:**
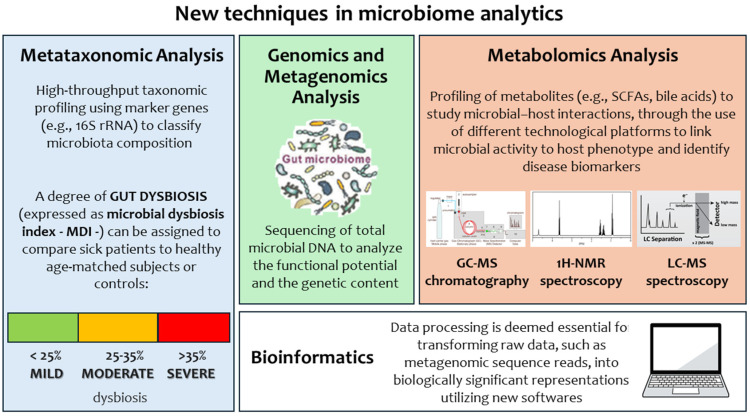
New techniques in microbiome analytics: metataxonomic analysis, genomics and metagenomics analysis, metabolomics analysis.

Metagenomics is currently the most investigated and, hence, the most advanced discipline able to design the correct microbial systems biology of the human body’s microbial consortia. Metagenomics, ranging from biodiversity metagenomics to meta-organismal metagenomics, covers almost all aspects of human physiology and pathology, with pioneering approaches for both data generation and handling. Metabolomic approaches have been widely applied in disease diagnostics: metabolic profiling can give an instantaneous snapshot of disease [136]. In this context, newborns with major CGISCs may be characterized by altered bacterial distributions of the fecal microbiota. A decrease in Bacteroides (Bacteroidetes) and *Bifidobacterium* (Actinobacteria) was registered in the fecal microbiota profiling of neonates with CGISCs [41]. Moreover, preterm surgical infants were characterized by an increase in Proteobacteria, *Staphylococcus*, *Enterococcus,* and *Klebsiella* [137,138] and a decrease in *Actinomyces* and *Corynebacterium* [139]. Using the metataxonomy method for the diagnosis of gut dysbiosis, patient GM profiles can be associated with a degree of microbial dysbiosis index (MDI) that can be assigned to the patient’s GM through a comparison of the GM profile to that of healthy age-matched subjects or controls, according to a study on pediatric inflammatory bowel disease [140] (Figure 1).

## 4. Discussion

This narrative review highlights the critical role of the GM in the health of neonates undergoing major surgery for CGISCs, a population particularly susceptible to dysbiosis. In a prospective cohort study, Rao compared stool microbiota composition and SCFA levels in 37 CGISCs newborns and in 36 healthy newborns. At week 2, CGISC newborns had a lower abundance of beneficial bacteria like *Bifidobacterium* and *Bacteroides* species, alongside an increase in opportunistic pathogens of genera Pseudomonas and Escherichia–Shigella. The stool concentrations of all SCFAs were lower in CGISCs [41] (Table 1).

This microbial imbalance is a consequence of prolonged antibiotic exposure, delayed enteral nutrition, and prolonged TPN [9,42]. Dysbiosis is associated with severe complications, such as NEC and sepsis [139].

Key strategies identified for promoting a healthier microbial environment include early initiation of HM feeding and careful antibiotic management, both of which are linked to improved microbial balance, as stated by the ERAS recommendations for perioperative care in neonatal intestinal surgery [58] (Table 1).

In a review with meta-analysis by Issac et al., EEN after gastrointestinal surgery reduced hospital stay and was associated with earlier fecal movements and reduced SSI without any increase in anastomosis leakage or gastrointestinal discomfort [51]. These results were in line with a previous meta-analysis by Behera evaluating EEN in children with bowel anastomosis [52] (Table 1).

In a prospective cohort of infants with CGISCs, early exclusive HM was associated with earlier full enteral feeding and reduced length of hospital stay [55] (Table 1).

Adjunctive therapies like probiotics and lactoferrin demonstrated potential efficacy for enhancing gut barrier function and immune modulation.

Trials with probiotic supplementation in newborns with CGISCs demonstrated their role in attenuating gut dysbiosis, but evidence is still limited, as reported in the systematic review by Rao et al. [59]. In their review, published in 2018, the authors analyzed data from two RCTs conducted by Powell and Murakami [60,61]. In a more recent RCT, the administration of a triple-strain probiotic containing a mixture of Bifidobacterium breve M-16V, Bifidobacterium longum subsp. infantis M-63, and Bifidobacterium longum subsp. Longum BB536 reduced the relative abundance of potentially pathogenic families and increased *Bifidobacteriaceae* [62]. Based on the evidence, a recent systematic review by Trivedi et al. underscores that probiotics in CGISCs show little or no effect on the incidence of sepsis or time to full enteral feeding but have an impact on GM composition, increasing beneficial bacteria [63] (Table 1).

Lactoferrin is useful in reducing LOS in preterm newborns, while data about its efficacy in reducing NEC are conflicting [128,129]. Regarding Lf use in postoperative management after gastrointestinal surgery, to date, there are no RCTs that assess its efficacy in CGISCs, as reported by Trivedi et al. [64] (Table 1).

### 4.1. Research Gaps

This review underscores the need for well-conducted larger-scale studies to confirm the efficacy of interventions like probiotics and lactoferrin in this category of infants with rare and heterogeneous conditions.

The questions of which probiotic strain to use, at which dosage, and the time of starting the treatment (preoperative or postoperative) are all issues that should be addressed in a well-conducted randomized trial [97]. Lactoferrin’s efficacy in modulating GM in surgical newborns should be evaluated in randomized trials, defining which product to use (bovin vs. human Lf), its purity and iron-binding capacity, and dosage.

There is currently no evidence available on the efficacy of synbiotics and postbiotics in the management of newborns needing surgery, but preliminary data on their potential effectiveness deserve to be evaluated in this setting of patients.

There is also a lack of standardized therapeutic protocols regarding the time to start enteral nutrition and time to reach full enteral feeding. This highlights the need for future research to focus on developing and validating these gaps in rigorous multicenter randomized controlled trials, which are difficult to perform due to the rarity of these conditions. Furthermore, while emerging metagenomic and metabolomic analyses are mentioned, their full potential in elucidating microbial dynamics in this specific patient group warrants further exploration.

### 4.2. Clinical Implications

**Figure 2:**
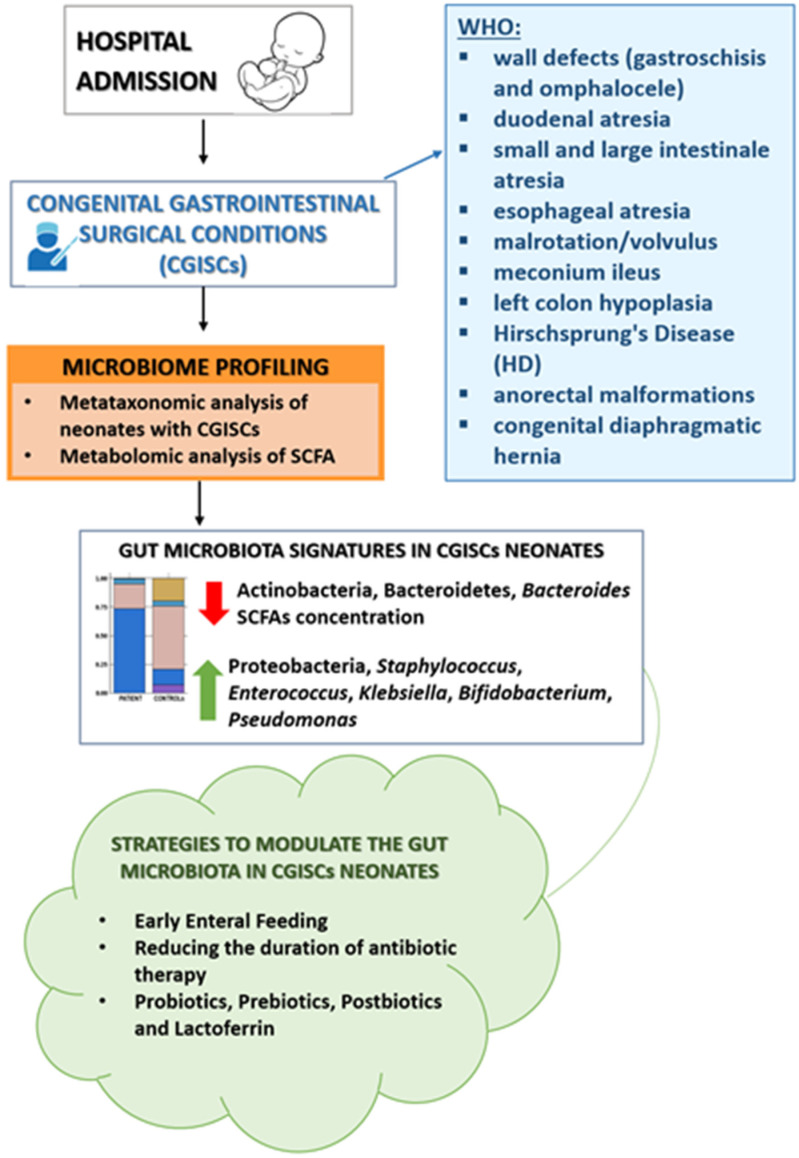
Flowchart of gut microbiome profiling to identify the GM signatures of CGICs in neonates and strategies to modulate their GM.

Our review suggests that proactive strategies to modulate the gut microbiota in neonates undergoing gastrointestinal surgery could significantly improve clinical outcomes. Children at risk of dysbiosis based on surgical pathology should be profiled with respect to the composition of their fecal microbiota and treated by adopting all the strategies that have proven to be most useful in counteracting the onset of dysbiosis, as proposed in Figure 2.

### 4.3. Limitations of the Review

This is not a fully systematic review; however, the process involved structured inclusion and exclusion criteria, a comprehensive literature search, and a critical appraisal of study quality. As a narrative review, a key limitation is its inherent subjectivity and potential for bias in literature selection and synthesis, which can influence a comprehensive representation of existing evidence. The flexibility of a narrative review allowed for broader discussions and the integration of different study designs, ensuring a well-rounded evaluation of strategies to modulate gut microbiota in neonates affected by surgical conditions.

## 5. Conclusions

Newborns with major gastrointestinal surgical conditions experience alterations of gut microbiota, with *Bifidobacteria* deficiency and a lower amount of SCFAs due to delayed enteral feeding, prolonged antibiotic course, parenteral nutrition, and exposure to the NICU environment. Microbiota alteration has been associated with poor feeding tolerance, sepsis, cholestatic jaundice, NEC, and poor neurodevelopmental outcomes. Strategies to modulate the GM are of utmost importance. Early nutritional interventions, especially the use of HM, and judicious antibiotic use should be prioritized. The potential benefits of some probiotic strains warrant consideration as supplementary treatments, though clinicians should be aware of the current limitations in their evidence-based management. Oral bovine Lf administration has been demonstrated to be useful in preterm newborns at risk of sepsis and to modulate gut microbiota and could therefore be a safe adjunctive strategy that deserves to be studied in newborns with CGISCs in large, well-conducted trials. Culture-independent metagenomic and metabolomic approaches to study microbiota composition and function will help understand the role of human microbiota in health and disease.

## Figures and Tables

**Table 2:** Key points and limitations for future research on probiotics in neonates who undergo major gastrointestinal surgery.

Key Points or Limitations	Details
Sample size	Many studies have a limited number of participants, which can reduce the statistical power and the generalizability of the results. The sample size should be calculated based on the result we aim to demonstrate, with a larger sample if we want to demonstrate a clinical benefit (e.g., reduction in mortality, NEC, LOS, time to reach full enteral feeding). Only large-scale studies can determine the benefits and risks of probiotic administration in these patients.
Heterogeneity of protocols	Differences in the probiotic strains used, therapy combinations, and definitions of primary outcomes can complicate comparisons between studies.
Randomization	Using “treatment allocation by minimization” or “rank minimization” could balance confounders such as mode of delivery and severity of the surgical condition.
Product safety and Quality	Probiotics available as dietary supplements may not meet the stringent safety, efficacy, and manufacturing standards required for pharmaceuticals.
Risk of infection	There is a potential (although very low) risk of sepsis associated with probiotic administration, especially in highly vulnerable neonates.
Challenges in studying gut microbiota	In neonates undergoing major surgery, surgical stress, inflammation, intravenous nutrition, and antibiotics can affect the gut microbiota, making it difficult to assess the effectiveness of probiotic administration. Stool samples should be collected and adequately stored for microbiota analysis and SCFA level measurements.
From bench to bedside	While probiotics may increase the proportion of “good” bacteria in the gut, it is unclear whether this translates into significant clinical benefits.

## Data Availability

Data for this narrative review have been reported from the available literature.

## Abbreviations

The following abbreviations are used in this manuscript:

NICU	Neonatal Intensive Care Unit
SCFAs	Short-Chain Fatty Acids
CGISCs	Congenital Gastrointestinal Surgical Conditions
TPN	Total Parenteral Nutrition
HD	Hirshsprung’s Disease
CDH	Congenital Diaphragmatic Hernia
NEC	Necrotizing Enterocolitis
PPIs	Proton Pump Inhibitors
H2RAs	H2-Receptor Antagonists
SBS	Short Bowel Syndrome
EOS	Early-Onset Sepsis
LOS	Late-Onset Sepsis
HM	Human Milk
DHM	Donor Human Milk
RCT	Randomized Controlled Trial
MDI	Microbial Dysbiosis Index
GM	Gut Microbiota
HMOs	Human Milk Oligosaccharides
GOSs	Galacto-Oligosaccharides
FOSs	Fructo-Oligosaccharides
bLf	Bovine Lactoferrin
Lf	Lactoferrin
ERAS	Enhanced Recovery After Surgery
GA	Gestational Age
PNALD	Parenteral Nutrition-Associated Liver Disease
BSI	Bloodstream Infections
LEN	Late Enteral Nutrition
EEN	Early Enteral Nutrition
SSI	Surgical Site Infection
TLR	Toll-Like Receptor
LPS	Lipopolysaccharides
MDRO	Multidrug-Resistant Organism
UTI	Urinary Tract Infection
FM	Formula Milk
